# Molecular differential diagnosis of follicular thyroid carcinoma and adenoma based on gene expression profiling by using formalin-fixed paraffin-embedded tissues

**DOI:** 10.1186/1755-8794-6-38

**Published:** 2013-10-07

**Authors:** Aleksandra Pfeifer, Bartosz Wojtas, Malgorzata Oczko-Wojciechowska, Aleksandra Kukulska, Agnieszka Czarniecka, Markus Eszlinger, Thomas Musholt, Tomasz Stokowy, Michal Swierniak, Ewa Stobiecka, Dagmara Rusinek, Tomasz Tyszkiewicz, Monika Kowal, Michal Jarzab, Steffen Hauptmann, Dariusz Lange, Ralf Paschke, Barbara Jarzab

**Affiliations:** 1 Department of Nuclear Medicine and Endocrine Oncology, Maria Skłodowska-Curie Memorial Cancer Center and Institute of Oncology, Gliwice Branch, Wybrzeże Armii Krajowej 15, Gliwice 44-101, Poland; 2 Clinic of Oncological and Reconstructive Surgery, Maria Skłodowska-Curie Memorial Cancer Center and Institute of Oncology, Gliwice Branch, Wybrzeże Armii Krajowej 15, Gliwice 44-101, Poland; 3 Radiotherapy and Chemotherapy Clinic, Maria Skłodowska-Curie Memorial Cancer Center and Institute of Oncology, Gliwice Branch, Wybrzeże Armii Krajowej 15, Gliwice 44-101, Poland; 4 Tumor Pathology Department, Maria Skłodowska-Curie Memorial Cancer Center and Institute of Oncology, Gliwice Branch, Wybrzeże Armii Krajowej 15, Gliwice 44-101, Poland; 5 Faculty of Automatic Control, Electronics and Computer Science, Silesian University of Technology, Gliwice Poland; 6 Division of Endocrinology and Nephrology, University of Leipzig Leipzig, Germany; 7 Department of General, Visceral, and Transplantation Surgery, University Medical Center of the Johannes Gutenberg University Mainz, Germany; 8 Genomic Medicine, Department of General, Transplant, and Liver Surgery, Medical University of Warsaw Warsaw, Poland; 9 Department of Pathology, Martin Luther University Halle-Wittenberg Halle (Saale), Germany

**Keywords:** Follicular thyroid adenoma, Follicular thyroid cancer, Gene expression, Microarray, Formalin-fixed paraffin-embedded blocks

## Abstract

**Background:**

Differential diagnosis between malignant follicular thyroid cancer (FTC) and benign follicular thyroid adenoma (FTA) is a great challenge for even an experienced pathologist and requires special effort. Molecular markers may potentially support a differential diagnosis between FTC and FTA in postoperative specimens. The purpose of this study was to derive molecular support for differential post-operative diagnosis, in the form of a simple multigene mRNA-based classifier that would differentiate between FTC and FTA tissue samples.

**Methods:**

A molecular classifier was created based on a combined analysis of two microarray datasets (using 66 thyroid samples). The performance of the classifier was assessed using an independent dataset comprising 71 formalin-fixed paraffin-embedded (FFPE) samples (31 FTC and 40 FTA), which were analysed by quantitative real-time PCR (qPCR). In addition, three other microarray datasets (62 samples) were used to confirm the utility of the classifier.

**Results:**

Five of 8 genes selected from training datasets (*ELMO1, EMCN, ITIH5, KCNAB1, SLCO2A1*) were amplified by qPCR in FFPE material from an independent sample set. Three other genes did not amplify in FFPE material, probably due to low abundance. All 5 analysed genes were downregulated in FTC compared to FTA. The sensitivity and specificity of the 5-gene classifier tested on the FFPE dataset were 71% and 72%, respectively.

**Conclusions:**

The proposed approach could support histopathological examination: 5-gene classifier may aid in molecular discrimination between FTC and FTA in FFPE material.

## Background

Discrimination between malignant follicular thyroid cancer (FTC) and benign follicular thyroid adenoma (FTA) is the most difficult aspect of thyroid pathology. Postsurgical (post-thyroid unilateral lobectomy) FTC and FTA management algorithms are different: only cancer patients require completion total thyroidectomy, adjuvant radioiodine treatment, and long-term follow-up. The histological diagnosis of FTC remains a challenge for pathologists, as the diagnostic criteria of FTC, namely capsular invasion or angioinvasion, are prone to serious inter-observer variability [1]. Thus, the discrimination of FTC from FTA is an important clinical problem, particularly for minimally invasive cases, and depends on the number of serial sections and tumour regions examined [2].

Several mutations play an important role in the biology of follicular tumours, such as *paired box gene 8 (PAX8)/peroxisome proliferator-activated receptor gamma (PPARG)* translocation and *RAS* point mutations. The first one occurs in 35–47% of FTC and up to 13% of FTA [3-5], the second occurs in approximately 20–50% and 19% of FTC and FTA, respectively [6-9].

Numerous single immunohistochemical markers have been proposed, and the most widely accepted single protein that improves diagnostic accuracy is galectin 3, even in the case of minimally invasive follicular carcinoma [10]. Obviously, immunohistochemical panels can be extended by including other proteins e.g., cytokeratin 19 or p27 [11]. Many other potential markers have been considered, such as HBME-1, extracellular matrix metalloproteinase inducer (EMMPRIN), growth arrest and DNA damage-inducible gene 153 (GADD153), thyroid transcription factor 1 (TFF-1), Ki-67, p63, and p53 [12-15], but the conclusions are limited by the relatively small size of tested populations.

Alternatively, the FTC and FTA differentiation problem was investigated in several miRNA-profiling studies. Some miRNAs are described as sensitive biomarkers of malignant and benign follicular thyroid tumours [16-18], but the overlap of differentiating miRNAs pointed out in these studies is limited.

A number of attempts have been made to improve the molecular diagnosis of FTC using specific mRNA signatures of malignant follicular tumours [19-26]. The most promising of these, by Borup and co-workers [19] was based on a 40-sample microarray dataset and led to the delineation of a 76-gene signature, which was highly sensitive and specific both for their own dataset and when tested with 2 previously published microarray datasets [21,24]. Simultaneously, a recent publication by Chudova et al. proposed a 167-gene classifier that was able to diagnose thyroid nodules with indeterminate cytology [27]. Although the classifier was trained on various types of thyroid benign and malignant nodules, the prospective multicentre validation study showed good specificity for follicular thyroid nodules [28]. The proposed predictors might be an important step to increase the effectiveness of thyroid nodule diagnosis. The vast majority of recent studies have concentrated on preoperative differential diagnosis [26,28], with molecular tests applied to fresh-frozen material with an aim to translate it into fine-needle aspiration biopsy specimen testing. This is evidently very important in the context of preoperative diagnosis, but it may not be the most efficient method for direct application to the analysis of formalin-fixed paraffin-embedded (FFPE) samples at the mRNA level, which in our opinion may aid postoperative differential diagnosis in controversial cases. In this study, we aimed to combine the gathered knowledge about the transcriptomes of FTC and FTA to derive a novel classifier of thyroid follicular malignancy applicable to FFPE material.

We therefore used the aforementioned available dataset of Borup *et al.* for gene pre-selection, further selected genes and trained the classifier on very carefully selected FTC and FTA samples, and verified the classifier by real-time quantitative PCR (qPCR) analysis in formalin-fixed paraffin-embedded (FFPE) samples.

## Methods

### Patients

Tumour samples for microarray analysis (fresh-frozen [FF] material) were derived from 27 FTC (median age, 68 years) and 25 FTA (median age, 47 years) patients treated by thyroidectomy in Polish and German centers. The study was approved by the local ethics committees of the MSC Memorial Cancer Center and Institute of Oncology (Gliwice, Poland), University of Leipzig (Leipzig, Germany), University of Halle (Halle, Germany), and Mainz University Hospital (Mainz, Germany) and informed consent was obtained from all the patients.

Tumour samples for further validation (FFPE blocks) were derived from 31 FTC patients (median age of patients, 61 years) and 40 FTA patients (median age, 49 years). All patients were treated by thyroid surgery at MSC Memorial Cancer Center and Institute of Oncology, Gliwice Branch, and samples were subjected to routine histopathological examination at the Department of Pathology between 2006 and 2010.

### Microarray analysis

To obtain the highest possible level of adequacy for histopathological diagnosis, samples containing enough tissue material were subjected to independent and blinded review by 2 thyroid pathology experts (S.H. and D.L.). Only samples with full assessment and concordant diagnoses from both pathologists were selected for the training dataset (hereafter “training dataset B”), which included 13 FTC and 13 FTA samples. The remaining samples were used as an independent set of samples (14 FTC and 12 FTA samples) constituting testing dataset D (Table 1), in which the initial clinical diagnosis was used to describe the sample. The term “training dataset” indicates the sample group used to build the gene classifier and the term “testing dataset” indicates the sample group used to test its performance.

**Table 1:** **Description of datasets analyzed in the study (see Additional file****1****for detailed information)**

**Dataset**	**FTC samples**	**FTA samples**	**Ref.**
Borup *et al.* microarray dataset A	18	22	[19]
Training microarray dataset B	13	13	this study
Validation qPCR dataset C	31	40	this study
Validation microarray dataset D	14	12	this study
Weber *et al.* validation microarray dataset E1	12	12	[24]
Hinsch *et al.* validation microarray dataset E2	8	4	[21]
Total	96	103	

RNA for microarray analysis was isolated using the RNeasy Mini kit (Qiagen, Hilden, Germany) after tumour content verification of the specimen by a pathologist. The standard Affymetrix microarray protocol was carried out, and samples were hybridized with the HG-U133 Plus 2.0 microarray. Microarray data analysis was performed in an R/Bioconductor environment. Datasets were pre-processed using the GC Robust Multiarray Average (GCRMA) method [29]. The classifier was developed using the CMA package [30]. Detailed methods are described in supplemental information (Additional file 1).

### Validation experiment in FFPE samples

Blocks with sufficient tumour tissue in the specimen (approximately 80%) were selected. RNA was isolated using the FFPE RNeasy Mini Kit (Qiagen) from 5 slices of paraffin blocks selected by a histopathologist. Details are provided in Additional file 1.

Real-time quantitative PCR (qPCR) was carried out for 8 genes selected from the training dataset: carbonic anhydrase IV (*CA4)*, engulfment and cell motility 1 (*ELMO1)*, endomucin *(EMCN)*, inter-alpha-trypsin inhibitor heavy chain family, member 5 (*ITIH5)*, potassium voltage-gated channel, shaker-related subfamily, beta member 1 (*KCNAB1)*, low density lipoprotein receptor-related protein 1B *(LRP1B)*, pleckstrin homology domain containing, family G (with RhoGef domain) member 4B (*PLEKHG4B)*, and solute carrier organic anion transporter family, member 2A1 (*SLCO2A1*). PCR amplification was performed with Universal Probe Library fluorescent probes (Roche, Basel, Switzerland) and a 5′-nuclease assay, starting with 200 ng of total RNA. Normalization was carried out in the GeNorm application [31]. Details of the methods are provided in Additional file 1.

### External microarray data and analysis pipeline

Three microarray datasets were downloaded from gene expression repositories: the datasets of Borup et al. (dataset A, 22 FTA and 18 FTC), Weber et al. (testing dataset E1, 12 FTA and 12 FTC), and Hinsch et al. (testing dataset E2, 4 FTA and 8 FTC). Details are provided in Table 1 and in Additional file 1.

**Figure 1:**
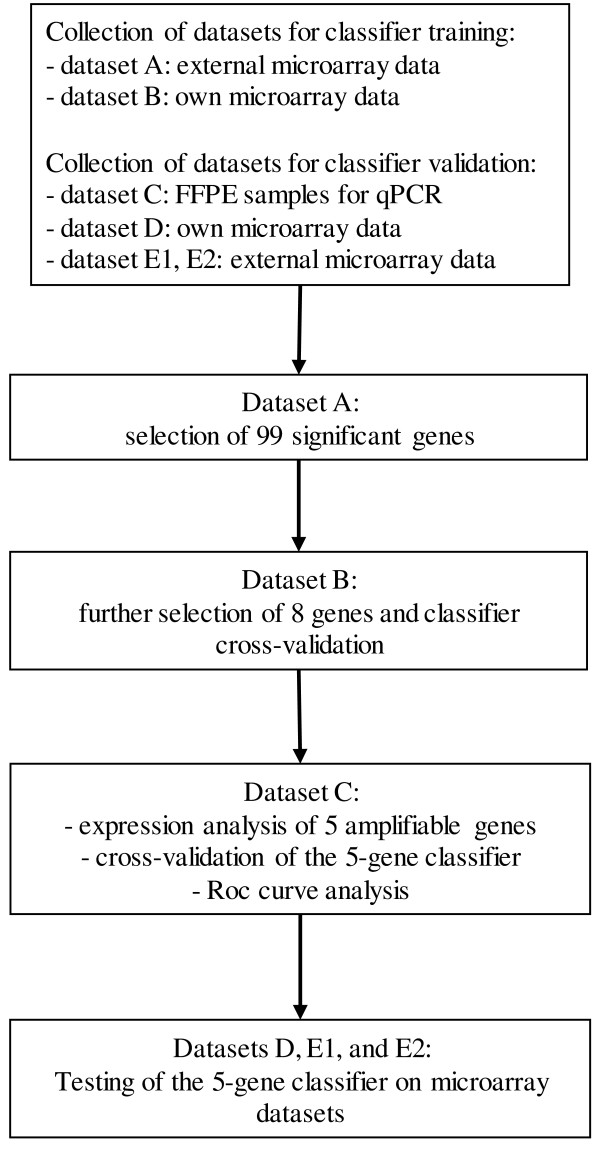
**Analysis pipeline.** First, datasets were collected. Second, the micorarray dataset A was analyzed and 99 genes were selected. Third, the microarray dataset B was analyzed for further selection of 8 genes and classifier cross-validation. Next, qPCR dataset C was analyzed in order to validate the classifier. Finally, public datasets D, E1 and E2 were analysed to test the classifier.

Dataset A was initially used for gene pre-selection. Further selection of genes and training of the classifier was carried out on dataset B (13 FTA and 13 FTC). The obtained classifier was tested in a group of independent new FFPE samples analyzed by qPCR (dataset C, 40 FTA and 31 FTC). Additional testing was carried out on microarray datasets D (our own samples, fresh-frozen, 12 FTA and 14 FTC) and the publicly available microarray datasets E1 and E2. In total, 199 thyroid samples were analyzed (123 of our own samples and 76 publicly available samples). The clinical characteristics of all specimens used in the analysis is presented in Table 1 and the analysis pipeline is described in Figure 1.

### Classifier construction and validation

Genes that exhibited significant differences between FTC and FTA were pre-selected from dataset A. The selection criteria were as follows: Student’s t-test (with equal variances assumed), non-corrected p-value <0.0005 (<0.09 when the p-value was corrected for multiple comparisons using the false discovery rate method), mean gene expression >5 in either the FTC or FTA group, and an absolute log ratio between the groups >1.5. Transcripts not fully annotated were filtered out. When more than one probe set per transcript was found, the probe set with the lowest p-value was selected.

Based on the subset of pre-selected genes, further gene selection using the Student's t-test (equal variances assumed), and classifier training were carried out on dataset B. Diagonal linear discriminant analysis (DLDA), a simple and reliable method based on linear combinations of genes, was chosen as the classification engine. However, other more sophisticated methods of classification were also used for comparison. Details of the analysis are provided in Additional file 1. The accuracy of the classification was assessed on dataset B using 10-fold cross-validation, repeated 10 times.

The 5-gene classifier was validated by qPCR on dataset C. First, the normalized expression data were log_2_-transformed. Then, the classifier performance was assessed using leave-one-out cross-validation (LOOCV; in each iteration, the DLDA classifier was trained on n-1 samples and tested on the remaining one). All 5 genes (validated by qPCR, details in results) from dataset C were used in each iteration and no further gene selection was used in the cross-validation loops.

Dataset C was also used to create a receiver operating characteristic (ROC) curve and to assess the diagnostic efficacy of the classifier. In the leave-one-out loop, for each sample, we calculated the probability that the sample belongs to the FTC class. Varying the threshold for the probability, the ROC curve was plotted.

An additional validation of the 5-gene classifier was performed using microarray data (datasets D, E1, E2). Both training dataset B and testing dataset D contained microarrays of the same type. Therefore, the final classifier, which had been trained on dataset B, was directly tested on dataset D. This approach was not possible for testing datasets E1 and E2 as they contained different microarray types. Therefore, LOOCV was used to assess the accuracy of the classifier on those 2 datasets. In all validation steps, only 5 genes amplified by qPCR were analyzed in the microarray test datasets and no further gene selection was used in the cross-validation loops.

### Comparison of the classifiers

To compare the 5-gene classifier developed by us to other published classifiers (the 76-gene classifier developed by Borup *et al.*[19], 3-gene classifier developed by Weber *et al.*[24], and 5-gene classifier developed by Foukakis *et al.*[32]), we calculated the accuracies of those classifiers in 4 different datasets: dataset B (our own), dataset D (our own), dataset E1 [24], and dataset E2 [21].

Borup’s classifier was originally created on dataset A and was based on 76 differentially expressed genes and the SVM method with a radial kernel. To calculate its accuracy on dataset B, we performed a 10-fold cross-validation of such a classifier with a fixed list of 76 genes. To calculate its accuracy on dataset D, we trained this classifier on dataset B and tested it on dataset D. The accuracy of this classifier on datasets E1 and E2 had already been reported by Borup *et al.* and we used those values for comparison.

To calculate the accuracies of the classifiers developed by Weber and Foukakis, we applied a DLDA classification method. Analogous to the analysis above, to calculate the accuracies of these classifiers on dataset B, we performed a 10-fold cross-validation. To calculate their accuracies on dataset D, we trained those classifiers on dataset B and tested on dataset D. To calculate their accuracies on dataset E1 and E2, we performed LOOCV analysis.

The accuracy of the 5-gene DLDA classifier on dataset B was calculated by 10-fold cross-validation (genes were selected by t-test). The calculation of the accuracy of our classifier on datasets D, E1, and E2 is described above.

## Results

### Developing a robust classifier for FTC and FTA differentiation

Because we aimed to obtain the most robust classifier possible, and had access to 2 reliable datasets (our own dataset with 26 samples [training dataset B] and the dataset of Borup *et al.* with 40 samples [dataset A]), we decided to use both of them sequentially for classifier construction. First, from dataset A (large enough to represent the variability of FTC) we selected 99 genes with high significance and large magnitude of difference (see Methods; the genes are listed in Additional file 2, and the raw expression values of these genes in dataset B are attached in Additional file 3). Second, based on the pre-filtered genes, we used dataset B (with histological diagnosis verification for each sample, based on the consensus diagnosis of 2 independent histopathologists) to build the classifier.

Cross-validation revealed that for the majority of classification engines used, there was no meaningful increase in multigene classifier accuracy, when more than 20 genes were used within the preselected dataset of 99 genes. (Additional file 1: Figure A). In an attempt to create a classifier of low complexity and due to the material limitation, we decided to validate only 8 genes, which provided almost the same accuracy as that for a larger number of genes: 80% and 84% accuracies for the 8-gene and 45-gene classifiers (the one with maximal accuracy), respectively (Table 2).

**Table 2:** Performance measures of classifiers in different datasets

**Dataset (origin, method of analysis)**	**Method**	**Accuracy (%)**	**PPV (%)**	**NPV (%)**	**Sensitivity (%)**	**Specificity (%)**	**Prevalence of FTC in dataset (%)**
B (own, microarray)	DLDA classification based on the 8 best genes chosen from 99 preselected ones.*	80	82	78	76	83	50
	DLDA classification based on 45 (optimal number) best genes chosen from 99 preselected ones.*	84	85	83	83	85	50
**C (own, FFPE qPCR)**	5-gene DLDA classification (cut-off 0.5)**	**72(95% CI: 60–82)**	**67(95% CI: 48–82)**	**76(95% CI: 60–89)**	**71(95% CI: 52–86)**	**72(95% CI: 56–85)**	**44**
	5-gene DLDA classification (cut-off 0.12)**	70	61	88	90	55	44
D (own, microarray)	5-gene DLDA classifier trained on dataset B, tested on D	73	77	69	71	75	54
E1 (Weber *et al.* microarray)	5-gene DLDA classifier.**	92	100	86	83	100	50
E2 (Hinsch *et al.* microarray)	5-gene DLDA classifier.**	83	100	67	75	100	67

Accuracy, proportion of all samples that are correctly classified; *PPV*, positive predictive value; *NPV*, negative predictive value; *SVM*, support vector machines; *DLDA*, diagonal linear discriminant analysis; *CI*, confidence interval. *Performance assessed by 10-fold cross-validation. **Performance assessed by leave-one-out cross-validation.

For the classifier, we selected 8 transcripts that were most significant in the analysis of 99 preselected genes on dataset B: *CA4*, *ELMO1, EMCN*, *ITIH5*, *KCNAB1*, *LRP1B*, *PLEKHG4B*, and *SLCO2A1*. The classifier built on these genes was referred to as the 8-gene classifier. Although we did not use information regarding the direction of the gene expression change, all of these most significant genes were downregulated in FTC. Detailed information about the significance of these genes in all the datasets used in the paper is included in Additional file 4.

### Classifier testing on FFPE material and qPCR

We assessed the expression of all the 8 selected genes in serial dilutions of calibrator RNA (mixture of excellent quality RNA from FTA and FTC). Because of the poor amplification of *LRP1B*, the gene was excluded from further analysis (insufficient qPCR efficiency). Next, the genes of the 8-gene classifier were analyzed in test dataset C (40 FTA and 31 FTC samples, RNA from FFPE blocks). *CA4* and *PLEKHG4B* did not amplify sufficiently in FFPE samples (*CA4* was amplified in 4 FTA and none of the FTC samples, and *PLEKHG4B* in 3 FTAs and 1 FTC samples; data not shown). This was probably due to the low constitutive expression of these genes or their high susceptibility to degradation (Additional file 1: Figure C). To verify this, we evaluated the expression of these genes by transcriptome sequencing of 2 FTCs and noted that *LRP1B*, *CA4,* and *PLEKHG4B* exhibited low abundance in FTC, compared to other genes (Additional file 1: Figure D). Considering the poor amplification of the 3 genes, we decided to proceed with the 5-gene classifier.

**Figure 2:**
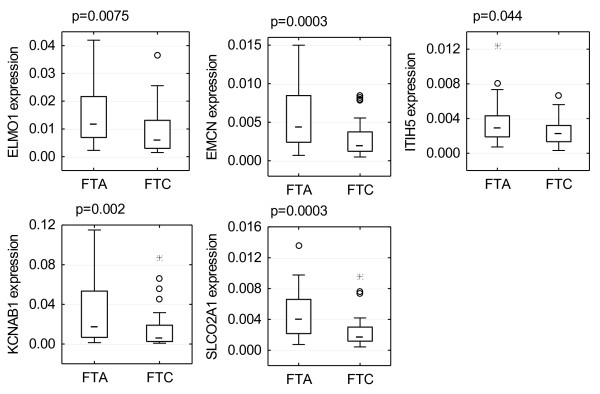
**Boxplots for the validated genes ( *ELMO1, EMCN, ITIH5, KCNAB1 *, and *SLCO2A1*).** All genes were under-expressed in follicular thyroid carcinoma (FTC) compared to follicular thyroid adenoma (FTA). All p-values were calculated using Mann–Whitney U test. The boxplots show following values: median: middle line; 25–75 percentile: box; non-outlying range: whiskers; outliers: circles; extreme values: stars.

We confirmed that *ELMO1*, *EMCN*, *ITIH5*, *KCNAB1*, and *SLCO2A1* were downregulated in follicular carcinoma compared to adenoma (Figure 2). The statistical significance of the differences for all 5 transcripts was assessed using the Mann–Whitney test. The results were significant for all of them when no correction for multiple comparison was used (p-value < 0.05), and for 4 of them (except *ITIH5*) when the Bonferroni correction was used (Table 3).

**Table 3:** Results for the 8 genes included in the classifier and chosen for qPCR validation on FFPE samples

**Gene**	**Signal log ratio (FTC vs. FTA)**			**Non-corrected p-value**	**Bonferroni-corrected p-value**
	**Dataset A**	**Training dataset B**	**Validation dataset C (FFPE)**	**Validation dataset C (FFPE)**	**Validation dataset C (FFPE)**
*ELMO1*	-2.15	-2.36	-0.98	**0.0075**	0.0377
*EMCN*	-1.94	-2.25	-1.17	**0.0003**	0.0015
*ITIH5*	-1.51	-1.98	-0.38	**0.0443**	0.2214
*KCNAB1*	-2.45	-3.35	-1.67	**0.0020**	0.0100
*SLCO2A1*	-1.86	-1.45	-1.23	**0.0003**	0.0014
*LRP1B*	-2.15	-2.7	Insufficient amplification		
*CA4*	-2.77	-2.92	Insufficient amplification		
*PLEKHG4B*	-1.88	-1.36	Insufficient amplification		

Log ratios of the 8 genes from microarray training datasets A and B, and validation dataset C (qPCR), are presented. Mann–Whitney U test results for genes validated with qPCR are in bold.

We tested the final 5-gene classifier on the qPCR/FFPE-derived test dataset C using a cross-validation approach and obtained a classification accuracy of 72%, sensitivity of 72%, specificity of 71%, positive predictive value (PPV) of 72%, and negative predictive value (NPV) of 67% (Table 2). Given that both PPV and NPV depend on the composition of the dataset, we also calculated the positive and negative likelihood ratios, which were 2.58 and 0.4, respectively.

**Figure 3:**
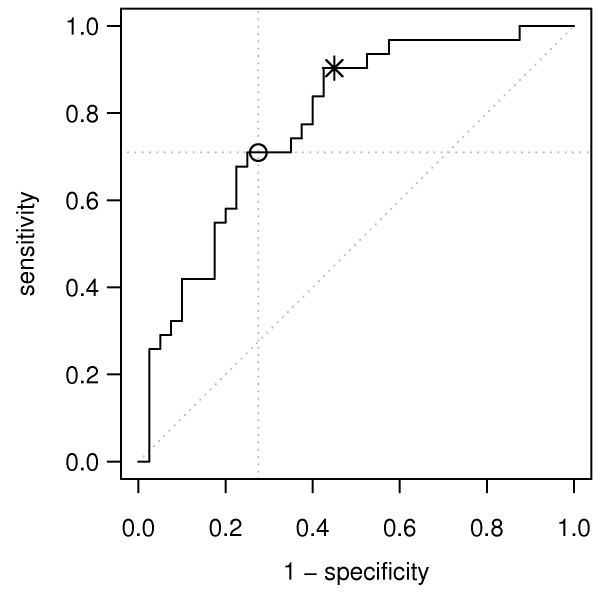
**ROC curve for the DLDA classifier that was cross-validated on the dataset C.** The circle marks the classifier with a cut-off of 0.5 (specificity = 71%, sensitivity = 72%). The star marks the classifier with a cut-off of 0.12 (specificity, 90%; sensitivity, 55%).

In our analysis, we treated the sensitivity and specificity as equally important. For diagnostic selection applications, however, maximizing sensitivity could be of greater importance, even if that decreases specificity. To analyse this aspect in-depth, we used the ROC curve. By shifting the 5-gene classifier probability threshold from 0.50 to 0.12, we were able to achieve a sensitivity of 90%, with some decrease in specificity (55%), and in the overall accuracy of the test (70%) (Table 2, Figure 3).

### Classifier testing on microarray datasets

The 5-gene classifier was further tested on 3 microarray datasets: D, E1, and E2. The classifier was trained on dataset B and tested on dataset D, resulting in an accuracy of 73%. Cross-validation of the classifier on datasets E1 and E2 provided accuracies of 92% and 83%, respectively (Table 2). These results further confirmed the reliability of the classifier.

### Comparison of the classifiers

We compared our simple 5-gene signature developed in combined analysis of dataset A and B to the complex 76-gene signature developed by Borup et al. on dataset A. We also compared our signature to the other classifiers, which are also composed of a small number of genes [24,32]. The accuracies of these classifiers, calculated on 4 datasets, are included in Table 4.

**Table 4:** Accuracy comparison for various classifiers

**Classifier description**	**Accuracy on dataset B**	**Accuracy on dataset D**	**Accuracy on dataset E1**	**Accuracy on dataset E2**
76-gene Borup's classifier	81%	81%	92%	83%
5-gene own classifier	77%	73%	92%	83%
3-gene Weber's classifier	77%	50%	91%*	42%
5-gene Foukakis classifier	77%	61%	71%	75%

*Potentially overfitting (tested on the set from which it was developed).

## Discussion

Although follicular tumours are routinely diagnosed post-surgically by classic histological criteria (tumour capsule infiltration and/or angioinvasion), this field of pathology is facing numerous challenges. These problems were recognized early but are still not fully resolved. A study from 1978 in Scandinavia has shown observer disagreement of nearly 30% when examining follicular carcinoma [33]. A report of agreement between 5 pathologists diagnosing follicular thyroid tumours reported their final consensus diagnosis in the range of 0.11-0.69 [1]. The inter-observer agreement for FTC diagnosis in that paper was estimated to 0.23, with large intra-observer variability (0.68) [1]. Similar data were obtained by our group [34]. This stresses the need for extensive training of pathologists involved in the diagnosis of follicular tumours, and the importance of reference centers, but also points to the necessity for tools that can improve pathologist accuracy. When mining for novel molecular markers, it is important that a consensus diagnosis by experienced pathologists be used, as was done in our work.

A number of gene expression profiling studies have attempted to identify transcripts that are differentially expressed between FTA and FTC [19-25]. However, none of these studies resulted in a simple, efficient gene signature that was applicable in clinical practice.

Recently, a promising gene expression classifier based on 167 genes was created [27] and validated [28] in a large multicentre trial. It was used for the discrimination between benign and malignant thyroid nodules that cannot be determined by cytology. It achieved a sensitivity of 92% and specificity of 52%, which is similar to our results (sensitivity 90% and specificity 55%). While these studies as well as other ones [28,35] lead towards a classifier applicable to fine-needle aspiration biopsy material, our approach is, in fact, different from these other published reports. We aim to support the diagnosis of post-thyroidectomy FFPE material carried out by routine histopathology with additional mRNA-based markers. Such a tool could be used as a pre-selection test to identify tumours that need meticulous histopathological evaluation. The genes selected in our study are differentiating in degraded mRNA specimens from FFPE blocks, and thus might potentially serve as a molecular indicator of malignancy. Both approaches (preoperative small sample cytology molecular testing [28] and post-operative whole section FFPE-based analysis, as proposed here) are in fact complementary and might be applied sequentially to provide the optimal final diagnosis.

In our opinion, it is necessary that the classifier be limited in the terms of genes tested, as cost-effectiveness issues may hamper the clinical application of multigene signatures; thus, selecting the optimal panel of markers for further testing is of utmost importance, especially from the perspective of health systems not reimbursing the cost of complex genomic diagnostic methods.

One of the important limitations of many previous studies concerning FTC/FTA differences is their relatively small sample size. To our knowledge, in all microarray studies published before 2010, the number of follicular tumours ranged from 7 to 28 [20,22]. The Borup *et al.* study, published in 2010, was based on a dataset of 40 follicular tumours, the largest non-custom microarray dataset of follicular tumours to date. Thus, the gene pre-selection in our study was based on a re-analysis of the Borup dataset.

Although our final 5-gene classifier is much smaller, it exhibits accuracy comparable to that obtained using the large, 76-gene classifier of Borup et al. On 2 of 4 datasets used for comparison, it gave the same accuracy as the large classifier, and in the other 2, the loss of accuracy was lower than 10%. When compared to other classifiers with a similar number of genes (3-gene classifier of Weber and 5-gene classifier of Foukakis), our classifier gives better accuracies on validation datasets. Our test set (dataset C), derived from FFPE samples and analyzed using qPCR, reproduces cases obtained in a routine setting. The classifier accuracy obtained on this dataset is 72%, with a sensitivity of 71% and specificity of 72%. For the detection of cancer, the sensitivity is of utmost importance; shifting the test threshold gives a sensitivity of 90%, with an acceptable specificity of 55%. This approach seems justified, as the gain in FTC diagnosis sensitivity would outweigh the drop in specificity via careful histopathological assessment.

From the initial 8-gene classifier, 3 genes: *CA4*, *LRP1B*, and *PLEKHG4B*, could not be amplified with qPCR, probably due to their low expression in FFPE specimens. The remaining 5 genes, positively validated in our analysis, are downregulated in FTC compared to adenoma. ELMO1, EMCN, KCNAB1, and SLCO2A1 are plasma membrane components and ITIH5 is an extracellular matrix (ECM) component. Some of these proteins exhibit functional similarities: SLCO2A1 and KCNAB1 exhibit transporter activity [36,37], and ELMO1, EMCN, and ITIH5 are involved in cell movement and ECM stability [38-40]. Some of the genes from our 5-gene classifier have already been mentioned in other studies of high-throughput gene expression analysis of follicular tumours. *ITIH5*, *KCNAB1*, and *SLCO2A1* are mentioned in the Borup et al. study. Besides, *ELMO1* is differentially expressed in Barden et al. [41] and *KCNAB1* is differentially expressed in 2 other papers by Takano [23] and Weber [24], respectively.

There are some potential limitations to our study. The most important issue, applying both to our analysis as well to all other studies carried out to date, is that we do not understand the reason for sample misclassification. Are the misclassified samples the same that would be misclassified by histopathologists? The excellent prognosis of follicular cancer treated with adequate surgical and adjuvant therapy makes this assessment difficult, given that patients exhibiting disease recurrence or dissemination initially or during follow up are relatively rare. This issue will be addressed in future studies, preferably using gene expression profiling of FTCs with metastases or local recurrence. We also cannot exclude the possibility that the reason for incorrect classification of some samples is that the signal from a small amount of neoplastic tissue might be dominated by the surrounding normal thyroid tissue.

Because routine diagnostic applications of the test is our goal, we did not apply any methods, such as paraffin slide micro dissection, to enrich the tumour content of our samples, as these methods are difficult to apply in the clinical setting. We believe that our signature is useful, as it was constructed using 2 large datasets and validated on an independent dataset using a different method. Although it is not efficient enough to be used as a clinical diagnostic test by itself, it would improve the process of diagnosis [42]. It is also a step towards developing a highly powerful classifier, as each microarray dataset generated and analyzed improves our understanding of gene expression differences between different types of follicular tumours. Additional studies also should be undertaken to elucidate the molecular and clinical aspects of the discovered markers.

We must stress that the problem of FTC and FTA differentiation has also been investigated by several miRNA-profiling studies. miRNAs are known to be a good disease markers, and can be easily detected in frozen tissues, paraffin blocks (FFPE), fine-needle aspiration biopsies (FNAB), or even serum [43]. Some miRNAs are described as sensitive biomarkers of malignant and benign follicular thyroid tumours [16-18]. In future, a possible FNA or FFPE-block test might combine degradation-resistant mRNA markers or protein markers and a panel of miRNAs to provide optimal analytical parameters.

## Conclusions

In summary, we developed a simple 5-gene classifier that can distinguish FTC from FTA with good accuracy. It is based on the 2 largest FTC-FTA microarray datasets and was validated on an independent FFPE sample set, with a potential sensitivity of up to 90%. In future, such a molecular test may serve as an important tool for assisting pathologists in cases of thyroid follicular neoplasms where a clear clinical decision cannot be made based on histopathology.

## Abbreviations

FTC: Follicular thyroid cancer; FTA: Follicular thyroid adenoma; qPCR: Quantitative real-time PCR; FNAB: Fine needle aspiration biopsy; FFPE: Formalin-fixed paraffin-embedded; FF: Fresh-frozen; GCRMA: GC robust multiarray average; DLDA: Diagonal linear discriminant analysis; LOOCV: Leave-one-out cross-validation; CA4: Carbonic anhydrase IV; ELMO1: Engulfment and cell motility 1; EMCN: Endomucin; ITIH5: Inter-alpha-trypsin inhibitor heavy chain family, member 5; KCNAB1: Potassium voltage-gated channel, shaker-related subfamily, beta member 1; LRP1B: Low density lipoprotein receptor-related protein 1B; PLEKHG4B: pleckstrin homology domain containing, family G (with RhoGef domain) member 4B; SLCO2A1: Solute carrier organic anion transporter family, member 2A1; PPV: Positive predictive value; NPV: Negative predictive value; ROC: Receiver operating characteristic; ECM: Extracellular matrix; PPARG: Peroxisome proliferator-activated receptor gamma; PDTC: Poorly differentiated thyroid carcinoma; PAX8: Paired box gene 8; EMMPRIN: Extracellular matrix metalloproteinase inducer; GADD153: Growth arrest- and DNA damage-inducible gene 153; TFF-1: Thyroid transcription factor 1

## Competing interests

The authors declare that they have no competing interests.

## Authors’ contributions

AP, BW, MJ, BJ contributed to the writing of the manuscript. MJ, ME, RP, and BJ designed and coordinated the study. AC and TM collected the tissue material. AK analysed patient data. ES, SH, and DL performed the histopathological examination of the samples. BW, MOW, DR, TT, MK performed the experiments described in this study. AP, TS and MS performed the bioinformatics analysis. RP and ME made critical revisions. All authors have read and approved the final manuscript.

## Pre-publication history

The pre-publication history for this paper can be accessed here:

http://www.biomedcentral.com/1755-8794/6/38/prepub

## Supplementary Material

Additional file 1 Description of data: This file contains additional detailed descriptions of Materials, Methods, and Results. Click here for file

Additional file 2 **Description of genes differentially expressed between FTC and FTA, derived in the analysis of dataset A.** Description of data: The table contains the list of 99 genes that exhibited significant difference between FTC and FTA in the analysis of dataset A. The table contains gene annotation information (affyids, symbols, names), analysis results (p-values and log-ratios calculated in datasets A and B), and information pertaining to which of the genes belong to the 5-gene, 8-gene, and optimal 45-gene classifiers. Click here for file

Additional file 3 **Expression values of genes differentially expressed between FTC and FTA, derived in the analysis of dataset A.** Description of data: The table contains the expression values of 99 genes that exhibited significant differences between FTC and FTA samples in the analysis of dataset A. The table presents their expression values in samples belonging to dataset B. Click here for file

Additional file 4 **Significance of the 8 selected genes in all analyzed datasets.** Description of data: The table contains the results of differential analysis for 8 selected genes in all analyzed datasets: A, B, C, D, E1, and E2. For each dataset and gene, it contains the p-value, fold change, log ratio, and the rank (according to the p-value) of the gene. Click here for file
