# Supplementary material for: Molecular differential diagnosis of follicular thyroid carcinoma and adenoma based on gene expression profiling by using formalin-fixed paraffin-embedded tissues
Source: BMC Med Genomics. 2013 Oct 7;6:38. doi: 10.1186/1755-8794-6-38 (PMC3852913; doi:10.1186/1755-8794-6-38)
Supplement: Additional file 1 — Description of data: This file contains additional detailed descriptions of Materials, Methods, and Results. [file 1755-8794-6-38-S1.pdf]

SUPPLEMENTAL INFORMATION

Gene expression microarrays

RNA isolation

Samples (40–150 mg) were ground in liquid nitrogen and homogenized in RLT Buffer (Qiagen) with beta-mercaptoethanol. RNA was extracted using RNeasy Midi Kit (Qiagen) and re-purified with RNeasy Mini Kit (Qiagen), including a digestion step with RNase-free DNase I set (Qiagen).

Microarray experiment

Five micrograms of RNA was used for cDNA synthesis. First, the RNA template was mixed, incubated with T7-oligo(dT)24 primer at 70 C for 10 min and placed on ice. Next, 5x First Strand Buffer (4 μL), dithiothreitol (2 μL; 0.1 M), and dNTPs (1 μL; 10 mM) were added and the mixture was preincubated at 42 C for 2 min. Then, Superscript II reverse transcriptase (1 μL; 200 U/mL) was added and the mixture was incubated at 42 C for 1 h. The mixture for second strand synthesis contained 30 μL of 5x Second Strand Buffer, RNase-free water (91.8 μL), dNTPs (3 μL; 10 mM), *Escherichia coli* DNA Polymerase I (4 μL; 10 U/mL), *E. coli* DNA Ligase (0.2 μL; 60 U/mL), and RNase H (1 μL; 2 U). After incubation at 16 C for 2 h, T4 DNA Polymerase I (2.5 μL; 4 U) was added and the mixture was incubated for 5 min at 16 C. The reaction was stopped using EDTA (10 μL; 0.5 M) and the double-stranded cDNA was purified with GeneChip Cleanup Sample Module (Affymetrix). All reactions were performed using One-Cycle cDNA Synthesis Kit (Affymetrix). For biotinylated cRNA synthesis, 7 μL of double-stranded cDNA were used (IVT Labeling Kit; Affymetrix). Labeled cRNA was purified with GeneChip Sample Cleanup Module and fragmented. The quality of cRNA was assessed with Bioanalyzer 2000, and ambiguous samples were first hybridized to a control microarray (Test 3), and then, after sample quality evaluation, to Human Genome U133 2.0 PLUS array (Affymetrix).

Washing, staining with streptavidin-phycoerythrin conjugate, and scanning of the arrays in a GeneArray 3000 scanner (Affymetrix) were performed as recommended by the Affymetrix Gene Expression Analysis Technical Manual.

External microarray data

Microarray dataset from the Borup *et al.* study (dataset A) was downloaded from the Array Express gene expression repository (http://www.ebi.ac.uk/arrayexpress/; accession number E-MEXP-2442). The dataset included various types of thyroid samples analyzed by HG-U133 Plus2 microarray. Only those relevant to our study were selected: 22 follicular adenomas and 18 follicular carcinomas. The data had already been preprocessed using GCRMA.

Raw microarray data from the Weber *et al.* study (testing dataset E1) was downloaded from the Array Express (E-MEXP-97). The dataset containing 12 FTC and 12 FTA was preprocessed using GCRMA.

Microarray data from the Hinsch *et al.* study (dataset E2) was downloaded from the Gene Expression Omnibus repository (http://www.ncbi.nlm.nih.gov/geo/query/acc.cgi?acc=GSE15045). The dataset containing 8 FTC and 4 FTA samples had already been preprocessed.

Quality assessment and pre-processing of microarray data (datasets B and D)

The quality of the microarray data was analyzed using arrayMvout [1] 1.12.0 library in R 2.14.1/Bioconductor environment. The data were pre-processed with the GCRMA method. Tumor samples were normalized together with normal thyroid samples that were not used in this publication.

Comparison of classifiers on the microarray training set (dataset B)

A variety of algorithms can be used to classify microarray data, but none of them is the best [2]. We used DLDA in our analysis, as this method is simple, reliable, and does not require parameter tuning. For comparison, we analyzed the accuracies of other classification methods. All the analyses were performed in the CMA 1.13.2 library in R 2.14.1/Bioconductor environment using our training

microarray dataset (set B). Only 99 genes chosen from Borup's dataset (set A) were analyzed using the following classification algorithms:

- KNN (K Nearest Neighbors) with different number of neighbors k = 1, 3, 5;
- DLDA (diagonal linear discriminant analysis);
- SCDA (shrunken centroid discriminant analysis, also called PAM);
- Support Vector Machines (SVM) with linear kernel;
- Support Vector Machines with radial kernel;
- Support Vector Machines with polynomial kernel;

We also used different number of genes (2, 3, 4, 5, 6, 7, 8, 9, 10, 12, 14, 16, 18, 20, 25, 30, 35, 40, 45, 50) to determine the effect of gene number on classification performance.

For each combination of classifier algorithm and number of genes, we applied the cross-validation procedure (10 loops of 10-fold cross-validation). In each iteration, a t-test was applied for gene selection. Next, the classifier was trained on 90% of the samples and then tested on the remaining ones. SVM parameters were tuned in the additional inner loop of the 5-fold cross-validation. For each combination of classifier algorithm and number of genes, we calculated the accuracy of the classifier and the misclassification rate. The results are shown in Figure A. Four algorithms, namely, DLDA, SCDA, linear SVM, and radial SVM, provided good accuracy. Up to 20 genes, the majority of classifiers showed a decrease of misclassification rate. Above 20 genes in classifier, for most of them the increase in number of genes did not result in improvement of accuracy.

Evaluating the influence of gene pre-selection on classification accuracy (dataset B)

In our analysis, we included a gene pre-selection step on microarray dataset A. To determine its effect on classification accuracy, we made a comparison. We omitted the pre-selection step and performed a 10-fold cross-validation repeated 10 times to assess the accuracy of classification performed on the entire dataset B. In each iteration, genes that were most significant in t-test were chosen. The analysis was performed for different number of genes (from 2 to 99). The same analysis was also performed

for dataset B using only the 99 genes that had been selected from set A. The results of the comparison indicate that inclusion of a pre-selection step significantly increases the accuracy of classification (Figure B).

Performance measures of classification

For each of the datasets, we calculated classifier performance measures, including accuracy (the proportion of all samples that are correctly classified), sensitivity (the proportion of FTC samples that are correctly classified), specificity (the proportion of FTA samples that are correctly classified), positive predictive value (PPV; the proportion of samples classified as FTC that are truly FTC), negative predictive value (NPV; the proportion of samples classified as FTA that are truly FTA), positive likelihood ratio (sensitivity divided by [1 - specificity]), and negative likelihood ratio ([1 - sensitivity] divided by specificity).

RNA isolation from FFPE samples (dataset C)

RNA was isolated using Qiagen RNeasy FFPE kit and resuspended in 20 µL of RNAse-free water supplied in the kit. RNA concentration was measured using the NanoDrop ND-1000 spectrophotometer. Reverse transcription was performed using Qiagen Omniscript RT Kit. The input RNA material for each sample was 200 µg in a final volume of 20 µL. A set of 71 samples (40 FTA vs. 31 FTC) were transcribed for qPCR experiment.

Calibration and standard curve preparation (dataset C)

For calibration and standard curve preparation, 6 high-quality RNA samples obtained from FF tumors (3 FTA and 3 FTC) were pooled together and RNA concentration was measured. A dilution series was prepared as follows: 1000 ng, 500 ng, 200 ng, 100 ng, 40 ng, 20 ng, 8 ng, and 2 ng. Additionally, 4 independent samples of 200 ng each of pooled material were used as calibrators. Serial dilutions, calibrators, and the 71 test samples were transcribed together in 1 RT-PCR run on a 96-well Biometra Uno II thermocycler. Subsequently all samples were diluted 10 times and distributed into qPCR

plates. Each plate comprised 71 experimental and 4 calibrator samples; additional plates were prepared for the standard curves.

Amplicon design for qPCR (dataset C)

qPCR settings were carefully designed to give the optimal amplification in FFPE-isolated RNA. General conditions for amplicon selection were:

- Short amplified fragments
- 3′-end proximity
- A common amplified sequence for all transcripts covered by the microarray probe
- Amplicon unique to human genomic and transcriptomic database (confirmed by BLAST)
- No known Single nucleotide polymorphisms (SNPs) in any of primer or probe sequence (BLAST SNP flanks)

We used the Roche Universal Probe Library for our experiments. Primers are listed in Table A:

QPCR experiment (set C)

All plates were amplified by 7900HT Fast Real-Time PCR on an Applied Bioscience machine. First, a standard curve was generated for each amplicon at each of the 8 concentrations (described earlier) in duplicate. The linear regression slope of the standard curve indicated amplification efficiency. Two independent plates were amplified for each gene. The GeNorm applet for Microsoft Excel was used to normalize qPCR data. Based on our experience with thyroid samples, we chose the EIF3A, EIF5, and HADHA genes for qPCR data normalization.

qPCR classifier equation

Based on the whole qPCR dataset C, we created a DLDA classifier formula, which can be used to classify new samples. The expression values are normalized, log-transformed (with base 2) and a value of 13 is added to all of them to avoid negative values.

classifier value = 0.587 * elmo1 + 0.891 * slco2a1 + 0.430 * kcnab1 + 0.839 * emcn + 0.570 * itih5

If classifier value is above 16.339, it denotes FTA, if it is below 16.339, it denotes FTC.

Discussion - description of the genes

SLCO2A1, commonly called prostaglandin transporter (*PGT*), is an organic anion transporter involved in prostaglandin transport [3]. Prostaglandins, produced by prostaglandin-endoperoxidase synthase 2 (PTGS2), have been linked to tumor progression, proliferation, and motility [4]. Down-regulation of *SLCO2A1* was confirmed in colorectal cancer [4]. To our knowledge, the importance of *SLCO2A1* in thyroid cancer has not been directly analyzed; however, in some studies, elevated expression of *PTGS2* in malignant cells was detected, whereas in other studies it was unaffected [5,6]. Prostaglandin regulation surprisingly links *PGT* to the orphan nuclear receptor peroxisome proliferator-activated receptor *gamma* (*PPARG*) [7]. PPARG prostaglandin-related ligands are transported by SLCO2A1 and therefore the physiological function of this nuclear receptor is dependent on PGT [7].

Another membrane transporter in our study is *KCNAB1*. Loss of its expression is linked with neuronal cell loss and neurofibrillary tangles in tauopathy brains [8] and it is a susceptibility gene for lateral temporal and focal epilepsy [9,10]. Although down-regulation of this transporter has been reported in thyroid cancer, validation results are contradictory [11]. Our study confirmed the down-regulation of *KCNAB1*.

ITIH5 is a member of a protein family that is commonly down-regulated in many types of cancers, leading to tumor progression [12]. In a recent study this gene was proposed as a potential prognostic marker for poorly differentiated thyroid carcinoma (PDTC) [13]. Our results are consistent with this finding [13]. Thus, *ITIH5* is a marker of a more aggressive histotype of follicular tumors and could therefore be a reliable indicator of malignancy. The ECM stabilization function of ITIH5 may explain its importance in the suppression of tumor progression.

EMCN was identified as an endothelial cell anti-adhesive protein that inhibits cell-ECM interactions [14]. Recent data showed that knockdown of EMCN inhibited cell growth and migration [15]. The down-regulation of endomucin observed in our study is inconsistent with these data.

ELMO1 participates in a Rac-dependent pathway of cell migration and phagocytosis [16]. Our results confirmed the down-regulation of *ELMO1* gene in FTC. ELMO1 protein has recently been shown to modulate PTGS2 activity in mesangial cells [17]. This observation may point to potential functional loops with PGT, which will be further investigated in FTC.

Supplemental Reference List

1. Asare AL, Gao Z, Carey VJ, Wang R, Seyfert-Margolis V: **Power enhancement via multivariate outlier testing with gene expression arrays.** *Bioinformatics* 2009, **25:**48-53.

2. Boulesteix AL, Strobl C: **Optimal classifier selection and negative bias in error rate estimation: an empirical study on high-dimensional prediction.** *BMC Med Res Methodol* 2009, **9:**85.

3. Lu R, Kanai N, Bao Y, Schuster VL: **Cloning, in vitro expression, and tissue distribution of a human prostaglandin transporter cDNA(hPGT).** *J Clin Invest* 1996, **98:**1142-1149.

4. Holla VR, Backlund MG, Yang P, Newman RA, DuBois RN: **Regulation of prostaglandin transporters in colorectal neoplasia.** *Cancer Prev Res (Phila)* 2008, **1:**93-99.

5. Garcia-Gonzalez M, Abdulkader I, Boquete AV, Neo XM, Forteza J, Cameselle-Teijeiro J: **Cyclooxygenase-2 in normal, hyperplastic and neoplastic follicular cells of the human thyroid gland.** *Virchows Arch* 2005, **447:**12-17.

6. Lee KJ, Jung YS, Kim WH, Yoon TI, Joo HJ, Soh EY: **Cyclooxygenase-2 expression in human thyroid disease.** *J Endocrinol Invest* 2008, **31:**111-118.

7. Nosjean O, Boutin JA: **Natural ligands of PPARgamma: are prostaglandin J(2) derivatives really playing the part?** *Cell Signal* 2002, **14:**573-583.

8. Wakasaya Y, Kawarabayashi T, Watanabe M, Yamamoto-Watanabe Y, Takamura A, Kurata T, Murakami T, Abe K, Yamada K, Wakabayashi K et al.: **Factors responsible for neurofibrillary tangles and neuronal cell losses in tauopathy.** *J Neurosci Res* 2011.

9. Busolin G, Malacrida S, Bisulli F, Striano P, Di BC, Egeo G, Pasini E, Cianci V, Ferlazzo E, Bianchi A et al.: **Association of intronic variants of the KCNAB1 gene with lateral temporal epilepsy.** *Epilepsy Res* 2011, **94:**110-116.

10. Cavalleri GL, Weale ME, Shianna KV, Singh R, Lynch JM, Grinton B, Szoeke C, Murphy K, Kinirons P, O'Rourke D et al.: **Multicentre search for genetic susceptibility loci in sporadic epilepsy syndrome and seizure types: a case-control study.** *Lancet Neurol* 2007, **6:**970-980.

11. Takano T, Miyauchi A, Yoshida H, Kuma K, Amino N: **High-throughput differential screening of mRNAs by serial analysis of gene expression: decreased expression of trefoil factor 3 mRNA in thyroid follicular carcinomas.** *Br J Cancer* 2004, **90:**1600-1605.

12. Hamm A, Veeck J, Bektas N, Wild PJ, Hartmann A, Heindrichs U, Kristiansen G, Werbowetski-Ogilvie T, Del MR, Knuechel R et al.: **Frequent expression loss of Inter-alpha-**

trypsin inhibitor heavy chain (ITIH) genes in multiple human solid tumors: a systematic expression analysis. *BMC Cancer* 2008, **8:**25.

13. Pita JM, Banito A, Cavaco BM, Leite V: **Gene expression profiling associated with the progression to poorly differentiated thyroid carcinomas.** *Br J Cancer* 2009, **101:**1782-1791.

14. Kinoshita M, Nakamura T, Ihara M, Haraguchi T, Hiraoka Y, Tashiro K, Noda M: **Identification of human endomucin-1 and -2 as membrane-bound O-sialoglycoproteins with anti-adhesive activity.** *FEBS Lett* 2001, **499:**121-126.

15. Kanki Y, Kohro T, Jiang S, Tsutsumi S, Mimura I, Suehiro J, Wada Y, Ohta Y, Ihara S, Iwanari H et al.: **Epigenetically coordinated GATA2 binding is necessary for endothelium-specific endomucin expression.** *EMBO J* 2011, **30:**2582-2595.

16. Gumienny TL, Brugnera E, Tosello-Trampont AC, Kinchen JM, Haney LB, Nishiwaki K, Walk SF, Nemergut ME, Macara IG, Francis R et al.: **CED-12/ELMO, a novel member of the CrkII/Dock180/Rac pathway, is required for phagocytosis and cell migration.** *Cell* 2001, **107:**27-41.

17. Yang C, Sorokin A: **Upregulation of fibronectin expression by COX-2 is mediated by interaction with ELMO1.** *Cell Signal* 2011, **23:**99-104.

18. Borup R, Rossing M, Henao R, Yamamoto Y, Krogdahl A, Godballe C, Winther O, Kiss K, Christensen L, Hogdall E et al.: **Molecular signatures of thyroid follicular neoplasia.** *Endocr Relat Cancer* 2010, **17:**691-708.

19. Weber F, Shen L, Aldred MA, Morrison CD, Frilling A, Saji M, Schuppert F, Broelsch CE, Ringel MD, Eng C: **Genetic classification of benign and malignant thyroid follicular neoplasia based on a three-gene combination.** *J Clin Endocrinol Metab* 2005, **90:**2512-2521.

20. Hinsch N, Frank M, Doring C, Vorlander C, Hansmann ML: **QPRT: a potential marker for follicular thyroid carcinoma including minimal invasive variant; a gene expression, RNA and immunohistochemical study.** *BMC Cancer* 2009, **9:**93.

Table A. qPCR primers

GENE	PROBE	FORWARD PRIMER	REVERSE PRIMER
CA4	#53	5′-tgtctaatatccccaaacctgag-3′	5′-ctccttggggagcaggtc-3′
ELMO1	#87	5′-gattccttgcaggacaaactg-3′	5′-agtcctttcccgtcaccac-3′
EMCN	#29	5′-ccatggaactgcttcaagtg-3′	5′-gaattattagctgcctctaaaacacc-3′
ITIH5	#69	5′-gagcattggggacatcca-3′	5′-gcacgaataccacattcttgg-3′
KCNAB1	#19	5′-tggggagcatcatcaagaa-3′	5′-tccaccccagtagagtttgg-3′
LRP1B	#76	5′-caaaagaacttacagagacccaca-3′	5′-cagcccaatatatgcgttca-3′
PLEKHG4B	#16	5′-agagccggagtctgtcctc-3′	5′-gccatgatgtgcctcagtc-3′
SLCO2A1	#19	5′-agttcttgttgatgcgcttg-3′	5′-aggagtggtcaatggtgagg-3′

Table B Clinical characteristics of samples

Dataset	Histology	Number of samples	Men (%)	Median age (years)	Ref.
Borup *et al.* microarray dataset (A)	FTC	18	39	65	[18]
	FTA	22	22	54	
Training microarray dataset (B)	FTC	13	38.5	67	this study
	FTA	13	0	41	
Testing qPCR dataset (C)	FTC	31	32	61	
	FTA	40	12.5	49	
Testing microarray dataset (D)	FTC	14	28.5	71	
	FTA	12	25	50.5	
Weber *et al.* testing microarray dataset (E1)	FTC	12	8	67	[19]
	FTA	12	unknown	unknown	
Hinsch *et al.* testing microarray dataset (E2)	FTC	8	37.5	49	[20]
	FTA	4	0	39	

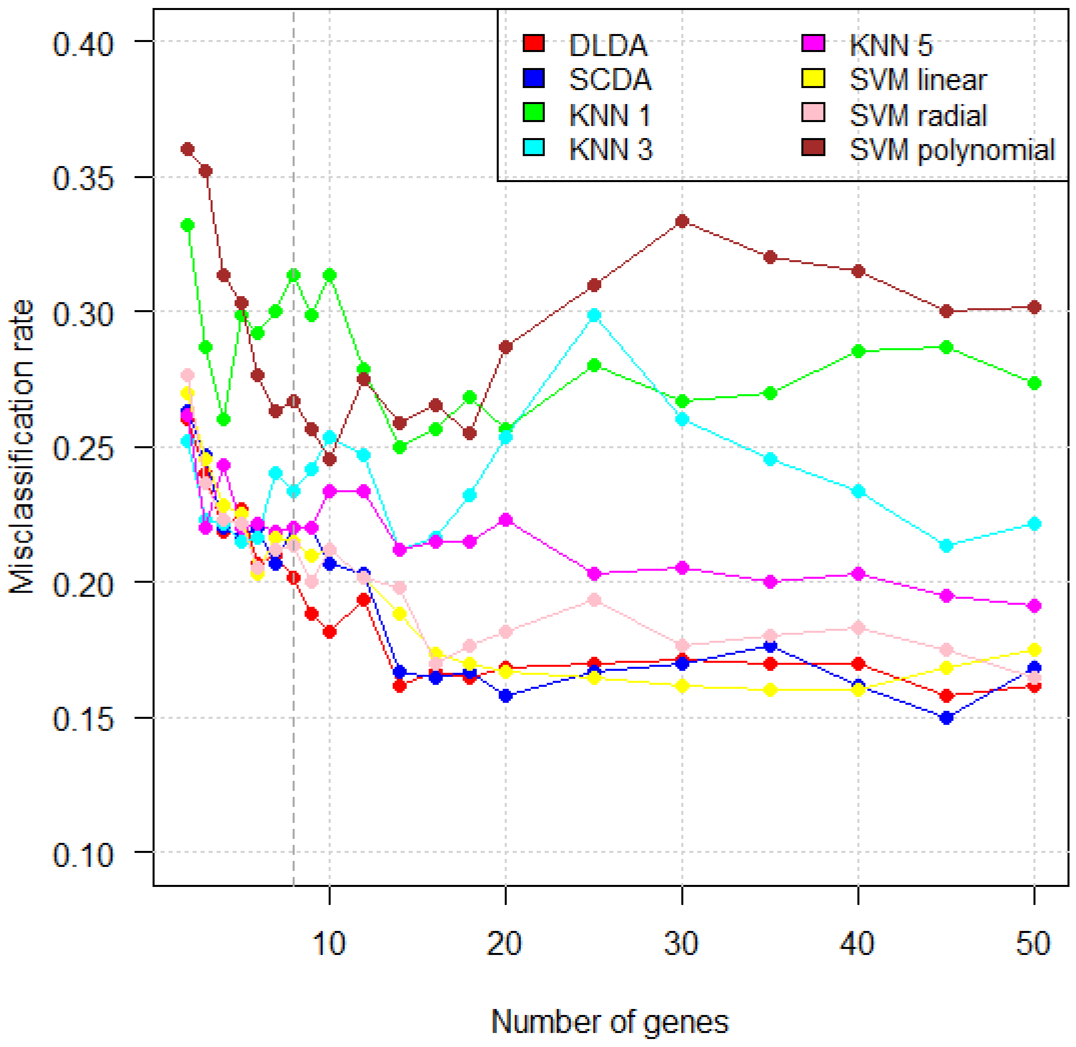

Figure A. Misclassification rates for various classification methods and different number of genes. The different colors indicate different classification algorithms. Misclassification rates were obtained on the training microarray dataset (B) in the cross-validation procedure. Abbreviations: DLDA - Diagonal Linear Discriminant Analysis, SCDA - Shrunken Centroid Discriminant Analysis, KNN 1 - K Nearest Neighbors with one neighbor, KNN 3 - K Nearest Neighbors with three neighbors, KNN 5 - K Nearest Neighbors with five neighbors, SVM linear - Support Vector Machines with linear kernel, SVM radial - Support Vector Machines with radial kernel, SVM polynomial - Support Vector Machines with polynomial kernel.

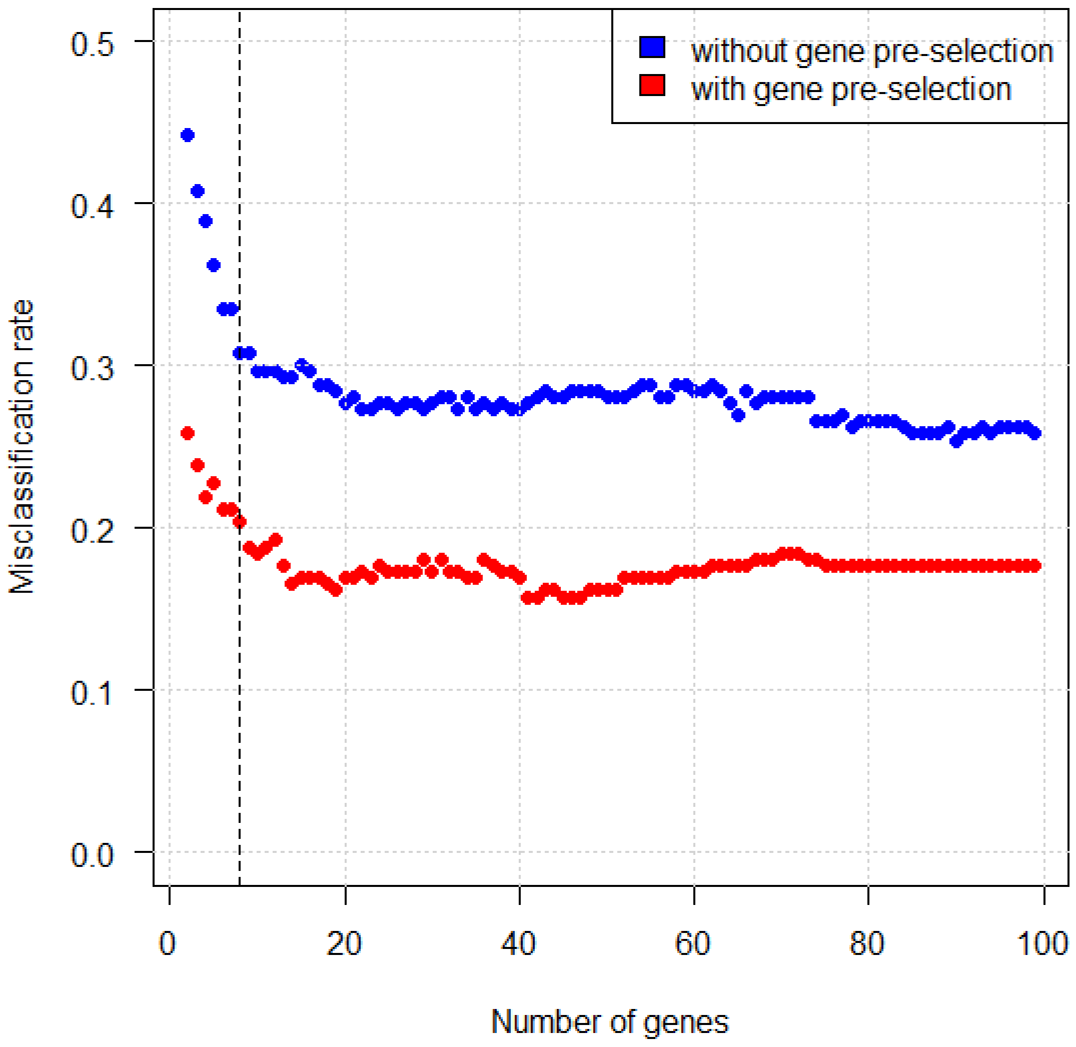

Figure B. Comparison of misclassification rates for the DLDA classifier on dataset B, with and without gene pre-selection on dataset A

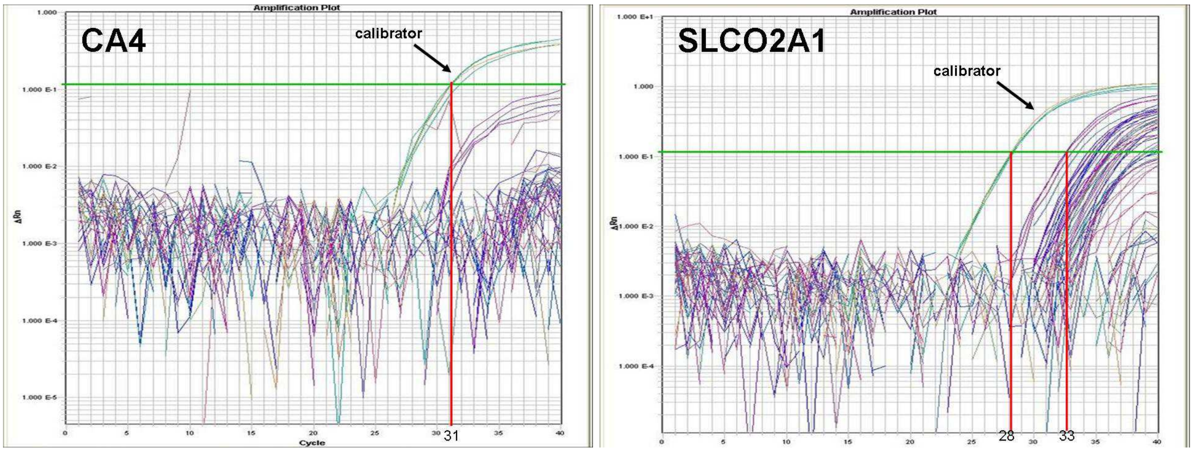

Figure C. qPCR amplification plots (set C). FF (fresh-frozen)-FFPE (formalin-fixed paraffin-embedded) comparison: qPCR amplification plots of *CA4* (Left) and *SLCO2A1* (Right). The Ct was significantly different between the FF (calibrator) and FFPE samples in both graphs. The best amplified FFPE samples exhibit a 5-cycle difference compared to frozen samples. *CA4* gene amplification curves for FFPE material were only obtained for 4/71 samples (all were FTA). Good amplification of the calibrator suggested that the designed amplicon was adequate and that the transcript levels in paraffin-derived samples were too low to be amplified.

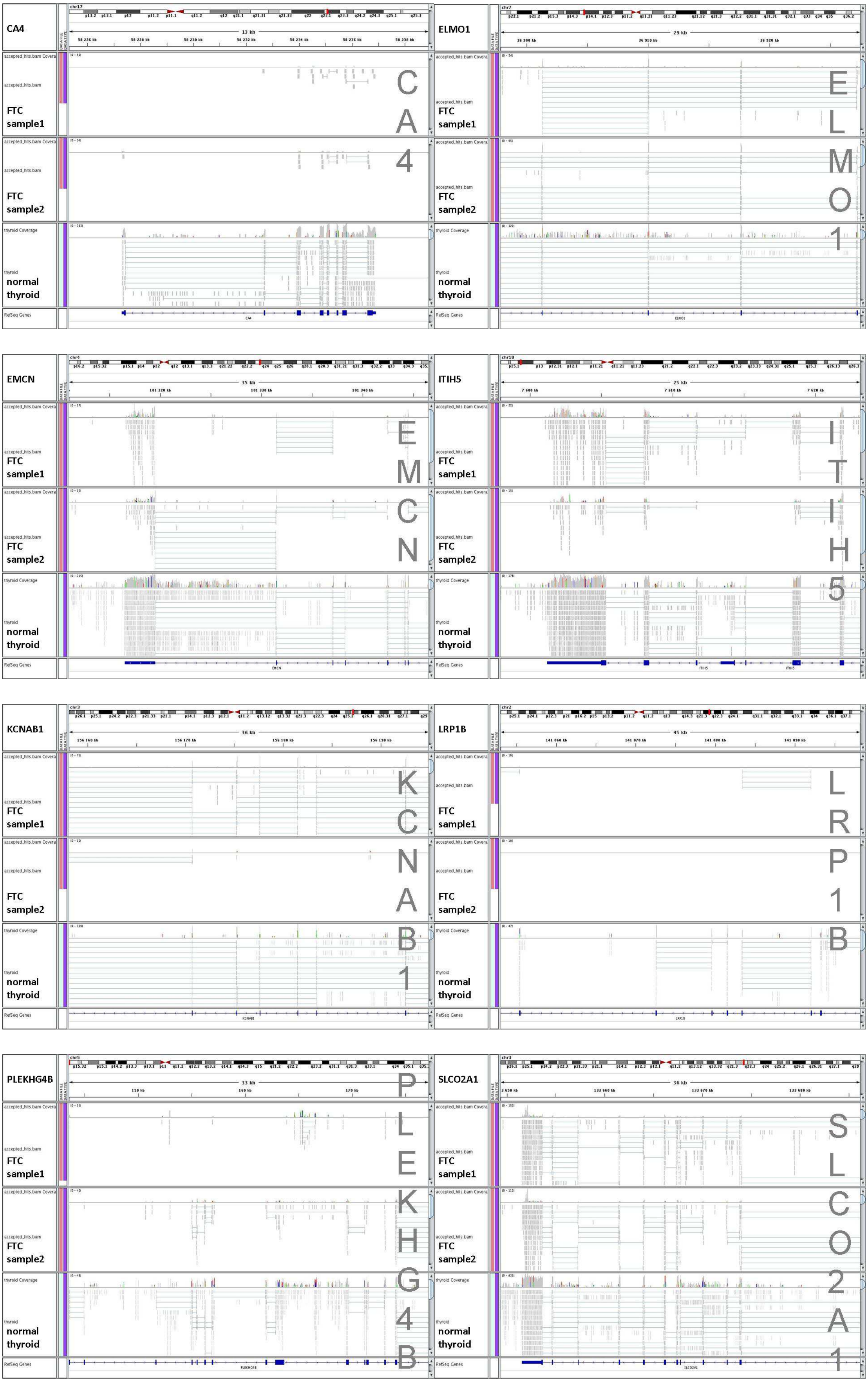
CA4
FTC sample1
FTC sample2
normal thyroid
RefSeq Genes
ELMO1
EMCN
ITIH5
KCNAB1
LRP1B
PLEKHG4B
SLCO2A1
C A 4
E L M O 1
E M C N
I T I H 5
K C N A B 1
L R P 1 B
P L E K H G 4 B
S L C O 2 A 1

Figure D. Next generation sequencing results. Eight transcripts from the classifier, visualized in IGV Genome Browser. The 2 upper lanes in each IGV snapshot are FTC samples and the lower lane is normal thyroid sample from Illumina Human Body Map 2.0 RNA-seq data. *CA4*, *LRP1B*, and *PLEKHG4B* have notably lower coverage than the other 5 genes.
